# Etching and Doping of Pores in Polyethylene Terephthalate Analyzed by Ion Transmission Spectroscopy and Nuclear Depth Profiling

**DOI:** 10.3390/membranes12111061

**Published:** 2022-10-28

**Authors:** Giovanni Ceccio, Jiri Vacik, Jakub Siegel, Antonino Cannavó, Andrey Choukourov, Pavel Pleskunov, Marco Tosca, Dietmar Fink

**Affiliations:** 1 Department of Neutron Physics, Nuclear Physics Institute (NPI) of the Czech Academy of Sciences (CAS), 250 68 Husinec, Czech Republic; 2 Department of Solid State Engineering, University of Chemistry and Technology Prague, 166 28 Prague, Czech Republic; 3 Department of Macromolecular Physics, Faculty of Mathematics and Physics, Charles University, V Holesovickach 2, 180 00 Prague, Czech Republic; 4 ELI —Beamlines Centre, Institute of Physics (FZU), Czech Academy of Sciences, 252 41 Dolni Brezany, Czech Republic

**Keywords:** membrane, ion-track etching, nuclear methodologies, pores

## Abstract

This work is devoted to the study of controlled preparation and filling of pores in polyethylene terephthalate (PET) membranes. A standard wet chemical etching with different protocols (isothermal and isochronous etching for different times and temperatures and etching from one or both sides of the films) was used to prepare the micrometric pores. The pores were filled with either a LiCl solution or boron deposited by magnetron sputtering. Subsequent control of the pore shape and dopant filling was performed using the nuclear methods of ion transmission spectroscopy (ITS) and neutron depth profiling (NDP). It turned out that wet chemical etching, monitored and quantified by ITS, was shown to enable the preparation of the desired simple pore geometry. Furthermore, the effect of dopant filling on the pore shape could be well observed and analyzed by ITS and, for relevant light elements, by NDP, which can determine their depth (and spatial) distribution. In addition, both non-destructive methods were proven to be suitable and effective tools for studying the preparation and filling of pores in thin films. Thus, they can be considered promising for research into nanostructure technologies of thin porous membranes.

## 1. Introduction

Polymeric membranes with etched ion tracks are known as polymer films with straight pore channels and have been investigated for decades and are still being studied today for their wide range of applications in applied and basic sciences [1,2,3,4,5]. A unique property of ion track membranes is that the pore density and pore diameter can be varied independently, which makes this polymeric membrane particularly interesting. An important aspect that must be thoroughly studied for this membrane is the full control of the membrane porous structure (i.e., the shape of the pores). Additional important properties that characterize the membrane are the transport properties and functionalization of pores by appropriate modification [6,7,8,9]. Different etching procedures allow for obtaining different pores geometries (cylinders, cones, double cones, cigar-like, and so on),; it has been shown that changes in the properties of membranes (e.g., diffusion and transport mechanisms, mechanical parameters, electric conductivity) can be achieved by variation of pores’ geometry, whereby during fabrication of the pores (using an adequate etching protocol) or by post-etching processing (e.g., thermal annealing), or also by inserting (and grafting) of specific materials into the pores [10,11,12]. Specifically, this last aspect is important for modern applications, such as fabrication of efficient catalysts or separators in Li-ion batteries [13,14]. For this reason, it is particularly important to have a full control over the pore shape during the preparation, and this can be achieved with adequate methodologies for the study of such shapes during different steps of the etching protocol. The importance of pore diameter is crucial for some properties of the membranes. The wettability [5], ability of liquid to maintain the balance between the intermolecular interactions of adhesive and cohesive types on the solid, the diffusion and separations of fluids [15], such as gas or liquid, the ion transportation phenomena [16], or the sensing ability [4] of functionalized membranes are characteristics strictly related to the pore shape and diameter.

Various sophisticated methods are used for the determination of structural parameters of nuclear pores. They are either non-destructive (e.g., X-ray and neutron diffraction) or invasive, damaging locally or in large areas the investigated polymers (e.g., SEM cross-section imaging, FIB-based techniques) [17,18]. Is well known that SEM studies provide highly accurate measurements of pore geometry and surface morphology; unfortunately, the method is limited in surface area and highly costly due to the equipment needed. Another method to determine the pore diameters is conductometry [19], which allows for determining the effective pore diameter by analyzing the voltage characteristic both after and during the etching process, but the disadvantage lies in the time-consuming process and sophisticated calculations that are model dependent. Recently suggested was the investigation of ion track membranes by non-contact ultrasonic spectroscopy [20], which takes advantage of the two propagation paths through membranes prepared with the ion track technique for the measurements of pore diameter and density. However, the procedure is time consuming and dependent on the mathematical model. For the purpose of this study, it was necessary to frequently analyze geometrical parameters of the pores during the etching process (at selected etching time) and monitor the influence of dopants, and the degree of their filling, on the shape of the pores. Therefore, it was necessary to use appropriate methods that would not damage the samples during repeated measurements, i.e., that would keep the geometry of the pores unchanged and allow several measurements with direct results. For this reason, ion transmission spectroscopy (with a low ion fluence) was chosen, which was able to analyze the 3D geometry of the pores, degree of filling with selected dopants, and also their effect on the pore shape. The depth (spatial) distribution of the defined material inserted into the pores (light elements —lithium and boron) was determined by neutron depth profiling. Both nuclear analytical methods are available in the equipment portfolio of the CANAM infrastructure at NPI Řež. In particular, the ITS method results in low cost with immediate interpretation of the filling degree of the membranes, based only on stopping power of the ions in the polymer. The aim of this study was to find out whether it is possible to follow the formation of the pores (and their shape) at different etching steps using a wet chemical etching method, and whether it is possible to fill the pores (and to what extent) with a material suitable for specific purposes, such as Li-ion batteries or targets for proton–boron fusion. For this project, it was important to provide suitable diagnostic methods that would allow for obtaining relevant information about the shape of the pores and their filling with selected material at various stages of etching.

## 2. Materials and Methods

### 2.1. Membrane Preparation

**Figure 1:**
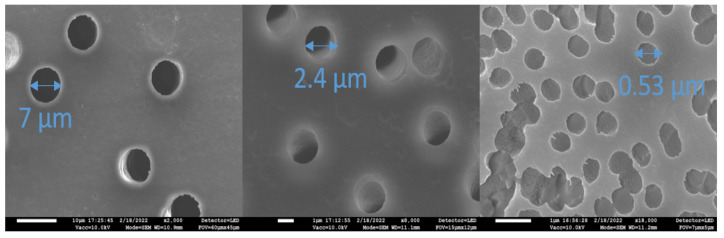
SEM micrographs of etched pores with diameters of 7.0 µm, 2.4 µm, and 0.53 µm.

Polymer membranes were prepared by wet chemical etching of ion tracks [21,22] in 19 µm thick polyethylene terephthalate (PET) films (provenance: Hostaphan^®^ Mitsubishi Polyester Films, Wiesbaden, Germany) irradiated by *Xe*^+^^26^ ions with an energy of 1.2 MeV/u (the ion irradiation of PET films was carried out at JINR Dubna by Dr. P.Y. Apel). Several different etching protocols were applied to prepare membranes with different geometries of pores:

(i) Asymmetric etching (i.e., one-side etching protocol) of the irradiated foils, performed consecutively, was applied to study the gradual development of pores under both isochronal and isothermal conditions. Different pore shapes were obtained for different etching temperatures and exposure times. For the etching procedure, a 9M NaOH solution was used in a temperature range of 55–75 C and etching times 0–60 min. For subsequent doping, 5M LiCl solution was selected as a dopant, and doping was carried out for 24 h at RT only from the side of etching at different stages of the pore development. After removing the sample from the dopant vessel, the sample surface was gently dried and cleaned from the excess dopant solution by wiping a smooth cloth over the sample surface.
(ii) In addition to one-side etching (performed in NPI Řež), a double-side etching protocol was also applied (JINR Dubna). Symmetric etching procedures made it possible to create membranes with cylindrical pores of several different diameters of 7 µm, 2.4 µm, and 0.53 µm (see Figure 1). Doping with boron was performed on only one side of the membrane (in addition to the PET films, a Si wafer was also used for the comparative analysis). Boron (99.9%, Kurt J. Lesker) was sputtered in Ar under a pressure of 5 Pa using a 3-inch planar magnetron powered by a radio frequency (13.56 MHz) power source. The power gradually increased (from 20 to 80 W) to avoid a thermal shock and cracking of the target. The total time of the deposition was 30 min, with a set thickness of 40 nm. Before deposition, the surface of the B target was pre-sputtered (cleaned) for 20 min.

### 2.2. Analytical Method

**Figure 2:**
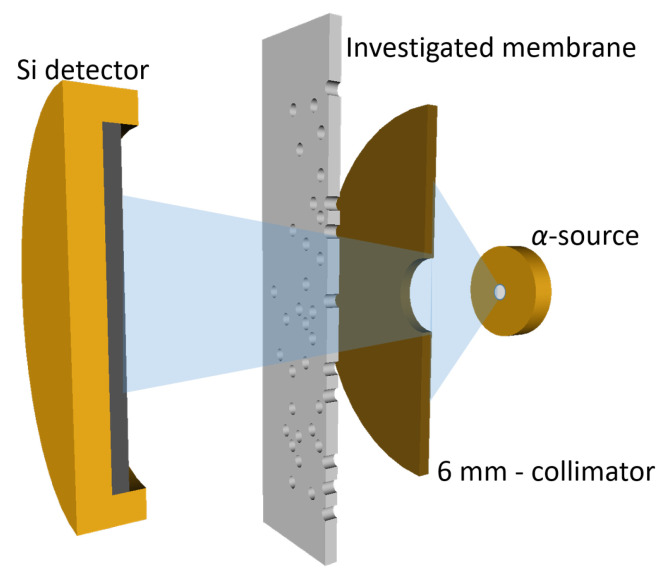
Ion transmission spectroscopy (ITS) setup assembled for the analysis of the PET membranes.

Specific nuclear analytical methods ITS and NDP, available in the NPI CANAM infrastructure [23], were applied to determine pore geometry and dopant incorporation in the PET membranes. Ion transmission spectroscopy (ITS) is a non-destructive nuclear technique based on the measurement of residual energies of MeV ions transmitted through thin films. It makes possible, by procession of the measured tomographic data, to determine the shape (3D geometry) of the micro-objects (e.g., etched pores or inclusions) in thin foils [24,25]. If ions pass through a porous membrane, they lose energy due to interactions with the polymeric material, and they are strongly affected by inhomogeneities (such as pores or dopants) along their path. Obviously, the transmission energy spectrum carries information about the average shape of the pores (and their areal density as well). If measured by a microbeam with a spatial resolution smaller than the pore size, the spectrum provides information on the shape of a single pore [26]. ITS sensitively reflects changes in the shape of pores, their areal density, and also their filling with other material. A thin, point-like ^241^ Am α-source with the main energy line of 5.486 MeV (82.7%) was used in this ITS study. It enabled the rapid and facile measurements of transmission spectra and evaluation of the instantaneous shape of the etched pores (Figure 2). The samples were measured in a small vacuum chamber, where the energy spectra were registered by a PIPS surface barrier detector, then stored in a TRUMP MCA and analyzed off-line using a Monte Carlo tomographic code (TOM), developed earlier by the author’s team [27].

**Figure 3:**
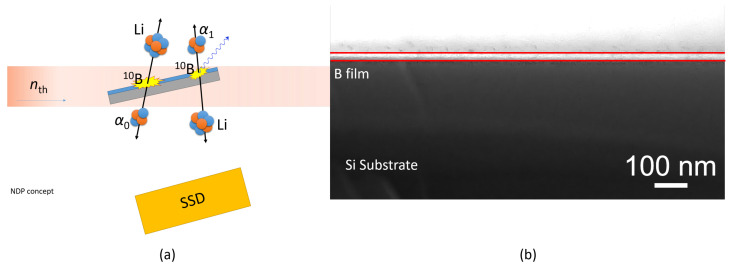
(**a**) Neutron depth profiling (NDP) setup, and (**b**) cross-section SEM of deposited B on the Si substrate.

The filling of the pores with boron was investigated by neutron depth profiling (NDP) [28,29]. It is a non-destructive technique utilizing high intense reactions with emission of charged particles to determine depth (spatial) distribution of the NDP-relevant elements. The method is based on the simulation of residual energies of charged reaction products registered by the NDP spectrometer and evaluated off-line by the MC code (LiBor), also developed by the author’s team [30]. In the case of boron, a nuclear reaction of ^10^B(*n*_th_, α)^7^Li was utilized with a high cross section of 3837 barns and relatively high reaction energy Q = 2.792 MeV. The NDP spectrometer is installed on the horizontal channel of the LWR15 research reactor (operated by the Research Centre Řež [31]); the NDP principle of operation is showed in Figure 3a. The samples were irradiated with thermal neutrons with a flux of 7 ×10^7^*n*_th_/cm^2^ provided by a supermirror neutron guide at a reactor power of 10 MW. The measurement was carried out in a large vacuum chamber with a background pressure of approx. 10^−^^1^ mbar. The samples were irradiated with a neutron beam at an angle of 15°; the solid angle (between the detector and the sample) was 0.382 × 10 ^−^^2^. Figure 3b shows a SEM cross-section image of the boron film with a thickness of ∼30 nm deposited on the Si wafer (JSM-7200F, Jeol Ltd., Tokyo, Japan).

## 3. Results

**Figure 4:**
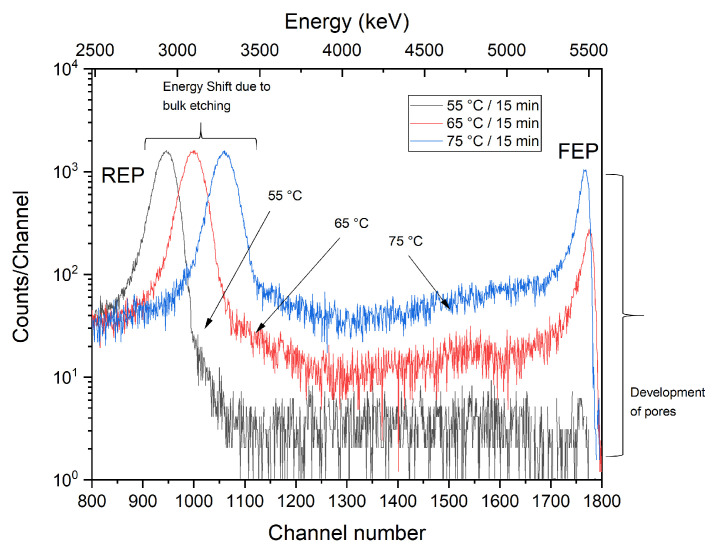
ITS results for porous membranes etched isochronously on one side of the PET foils for 15 min with different etching temperatures of 55 °C, 65 °C, and 75 °C.

Figure 4 shows a series of ITS spectra recorded on the PET membranes etched isochronously in 9M NaOH, i.e., for 15 min at several selected etching temperatures (55 °C, 65 °C, and 75 °C).

**Figure 5:**
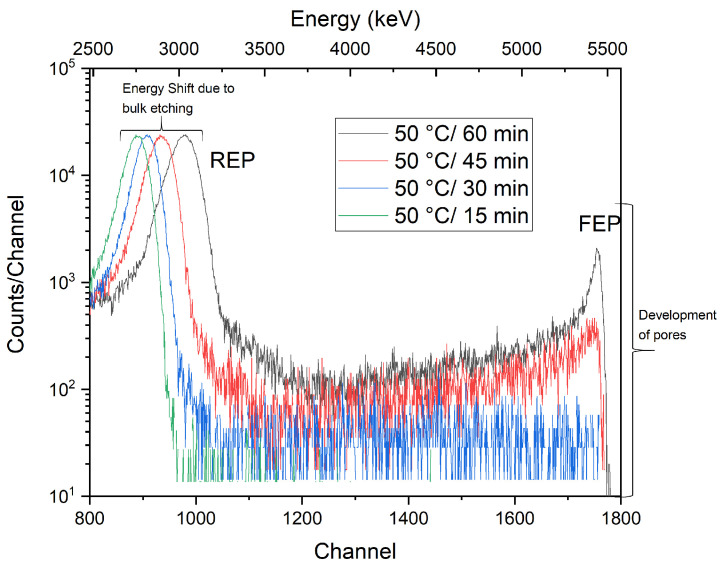
ITS results for porous membranes etched isothermally at 50 °C on one side of the PET foils with different etching times of 15, 30, 45, and 60 min.

As can be seen, the full energy peak (FEP, with a nominal alpha energy of 5.486 MeV) increases rapidly at higher etching temperatures, obviously because the etch rate of the latent tracks grows with the increasing temperature of the etchant and so does the size of the pores. In addition, the reduced energy peak (REP) shifts significantly to the higher energies (the reduced energy peak represents alpha particles passing through the unirradiated area of the membrane, i.e., with maximum energy loss). The shift is due to the reduction of the thickness of the membranes as a result of the etching of the non-irradiated area in PET. The ITS analysis of the transmission spectra in Figure 4 shows a sharp growth in the pore volume network (a spatial part of the membranes occupied by empty pores), which increased sharply (almost 25×) when the etching temperature changed from 55 °C to 75 °C. Pore volume growth is shown to be approximately linear in a given 15 min’s isochronous etching (except for slightly slower growth at a lower temperature of 55 °C) with a steep gradient (slope) of ∼2.8 (°C^−^^1^). Such a rapid change in the membranes’ porosity is to be expected; the etching conditions are harsh, and so the latent tracks can be quickly dissolved and removed. However, it is important that the etched pore walls are fairly smooth without some rough morphology. A finer etching was also carried out (under milder conditions with a lower etchant molarity and a lower etching temperature), which however meant a longer etching time but better control of the pore shape as needed. Figure 5 shows ITS spectra for the isothermal pore etching. The PET film was exposed to 9M NaOH at a fixed etching temperature of 50 °C for different times of 15, 30, 45, and 60 min. In this part of the experiment, a lower etching temperature was chosen to allow better sensitivity in controlling the evolution of the pore shape.

**Figure 6:**
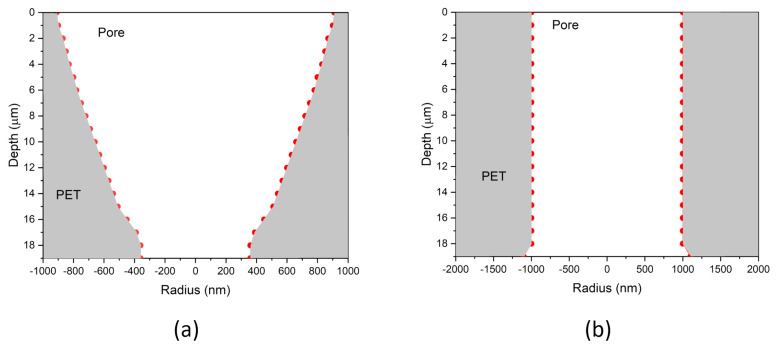
Pore shapes obtained by the TOM code simulation of the ITS spectra measured for the PET membranes etched isothermally at 50 °C for 45 min (**a**) and 60 min (**b**).

As can be seen, the transmission spectra record a similar course as in Figure 5 —that is, a sharp increase in the FEP peak, as well as the whole spectral area above REP, which means a dramatic evolution in the pore volume network. The analysis of the transmission spectra showed that, similar to the isochronous etching, the growth of pore volume is more or less linear with a very high slope of ∼5.1 (min^−^^1^) (except for the first 15 min etch). From the reconstruction of the spectra, evaluated by the TOM code, it was found that the breakthrough of the foil occurred just before 45 min of etching; the pores first acquired a conical shape, which gradually developed into a cylinder form after 60 min of etching. The two obtained profiles, calculated from the TOM code, are shown in Figure 6 (to be noted —the ITS spectra represent the averaged values of ion transmission data, so the TOM simulation gives an averaged form of individual pore shapes, where small irregularities are smoothed out). The obtained pore diameters are also in good agreement with literature value obtained with similar etching conditions and obtained with SEM microscopy [32,33]. These two pore shapes were then selected for the dopant incorporation and encapsulation tests.

**Figure 7:**
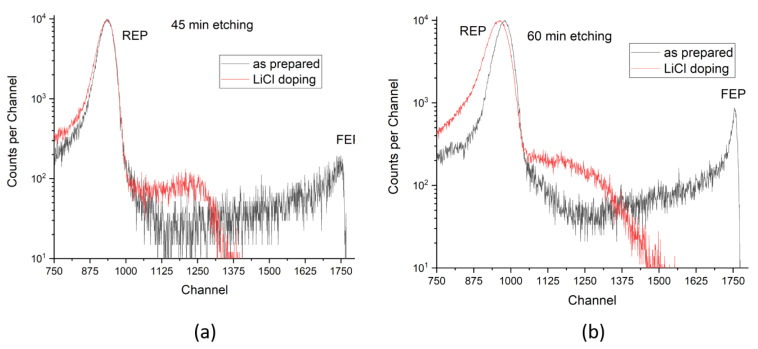
Comparison between the ITS spectra before LiCl doping (black line) and after LiCl doping (red line) for the PET membrane etched for 45 min (**a**) and 60 min (**b**).

Figure 7 shows the effect of incorporating 5M LiCl dopant into both pore types performed under the same conditions (doping only from the etched-side of the membrane for 24 h at RT). As can be seen, LiCl was effectively encapsulated in both types of pores (either due to ^7^Li capture by the electronegative ester groups -COOR exposed by etching on the pore walls, or simply in the form of loosely packed LiCl salt crystals trapped in the confined pore space), which completely closed their free passage of the probing alpha particles (the FEP area disappeared). On the other hand, a part of the spectra between REP and FEP increased, differently for both types of pores, which implies that the LiCl dopant is distributed in both pores in somewhat different ways.

From the ITS spectra, it can be concluded that the encapsulated LiCl substance does not form a compact structure (otherwise the spectrum above the REP would fully disappear), but acquires a somewhat dispersed form with a reduced average volume density, as compared with the textbook value of compact LiCl (2.07 g/cm^3^). This reduction amounts to approximately 24% for the narrower pores etched for 45 min, passing from a calculated free volume of 59.66 × 10^9^ nm^3^ to a free volume of 53.09 × 10^9^ nm^3^, and to approximately 11% for the larger pores etched for 60 min, with a reduction of free volume from 25.45 × 10^9^ nm^3^ to 19.342 × 10^9^ nm^3^, possibly pointing at a reduced accessibility for dopants in narrower pores. With an increasing etched pore size, the maximum energy of the transmitted alpha particles increased, indicating that the surface cleaning procedure may have removed some of the initially present near-surface LiCl precipitates in the pores. The slight broadening of the REP peak of the doped sample in Figure 7b suggests that some traces of the dopant solution still survived the surface cleaning in this case, eventually due to stronger surface adhesion, by profiting from an enhanced surface roughness of this longer-etched sample.

**Figure 8:**
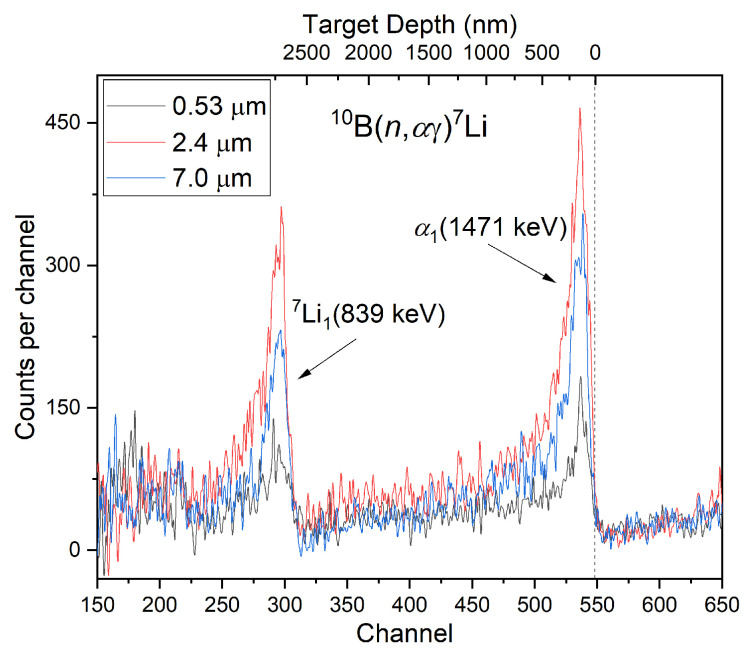
NDP spectra with boron sputtered on one side of a PET membrane with pores diameters of 7 µm, 2.4 µm, and 0.53 µm.

For a more accurate quantification of the concentration and spatial distribution of the dopants (made up of some light elements), the method of neutron depth profiling can be used. In the final part of the study, the membranes (with pores of different diameters) were doped with boron sputtered on only one side of the double-etched membranes. The aim was to determine the efficiency of doping of pores with a different size. Three membranes with pore diameters of 7.0 µm, 2.4 µm, and 0.53 µm were prepared. The NDP analysis was carried out from the opposite side of the membranes to which boron was deposited (as shown in Figure 3a) to avoid measuring of boron from the membrane surface. Therefore, only boron from pores could be analyzed. Boron (^10^B∼19.8%, ^11^B∼80.2%) was chosen as a marker because of the high effective cross section (3837 b) of the reaction ^10^B(*n*_th_, α)^7^Li with thermal neutrons. Figure 8 shows the NDP spectra obtained for all three boron-doped membranes with different pore sizes.

The spectra were normalized to the fluence of the neutron monitor (because of some variability of the LWR15 reactor power, it is needed to relate all NDP measurements to the fluence of the neutron flux, measured by a special spectrometer line). Monitoring the neutron fluence of the NDP measurements made possible an accurate analysis (concentration and depth distribution) of the boron dopant trapped in the pores. The peaks at channel 550 and 300 represent alpha and lithium particles, with full energy of 1.471 MeV and 839 keV, respectively, from the reactions generated in the pores. The spectra between the channels 550 and 300, utilizing only alpha particles, corresponds to an accessible depth of about 2.4 ≠ m for the NDP analysis. The following concentration of boron (^10^B) in pores with different diameters was determined based on the comparison with the NIST standard: 4.39 × 10^14^ cm^−^^2^, 5.96 × 10^14^ cm^−^^2^, and 2.20 × 10^14^ cm^−^^2^ for pores with a diameter of 7 µm, 2.4 µm, and 0.53 µm, respectively. Measured values correspond to films of B attached to the pore walls (close to their openings) with an average thickness of several tens of nm: ca 5.1 nm for pores 2.4 µm, ca 3.8 nm for pores 7 µm, and 1.9 nm for pores 0.53 µm. Interestingly, boron is most easily trapped in pores (near the opening of the con-side of the membrane) with a 2.4 µm diameter, which points —as expected —at higher adhesion (and reduced surface mobility) of adsorbents to surfaces with larger curvature.

## 4. Conclusions

The ion transmission technique ITS with 5.486 MeV alpha particles was used to investigate the etching process in PET films irradiated with 1.2 MeV/u *Xe*^+^^26^ ions. Pore shape development, their filling with LiCl and B dopants, as well as the etching rate of the non-irradiated areas were studied for both isochronous and isothermal etching conditions. The ITS study showed that even in a simple arrangement with an α-source, it was possible to monitor and quantify the evolution of the etched micron-size pores: their shape transferred from latent tracks to a conical form (for one-side etching), and through a symmetrical (double-side) etching process to a well-developed cylindrical geometry; the pore volume network increased approximately linearly with both etching temperature (with a slope ∼2.8 (°C^−^^1^) for etching time 15 min in a temperature range 55–75 °C) and etching time (with a slope ∼5.1 (°min^−^^1^) for etching temperature 50 °C and exposure time range 15–60 min); bulk etched rate for isothermal etching (at 50 °C) was determined as ∼24 nm/min. Doping the etched tracks with 5M LiCl solution resulted in a reduction of the pore volume network by 24% and 11% for etching times of 45 and 60 min, respectively. Together with ITS, a method of neutron depth profiling was also applied (which is very suitable for in-depth profiling of several light elements that cannot be determined easily by other techniques). The NDP method enabled non-destructive analysis (concentration and depth distribution) of boron with which the PET membrane surface and etched circular pores were covered and filled, respectively. It turned out that boron deposited by magnetron sputtering penetrated easily into the pores and diffused to their opposite side, where it attached mostly near their openings. Interestingly, the most boron was found in pores with a diameter of 2.4 mm. This study showed that using traditional wet chemical etching, controlled by the ITS method, it is possible to develop pores with a defined structure and to fill them in a controlled manner with a relevant light material whose spatial distribution can be determined by the NDP method. Thus, both non-destructive nuclear techniques have proven to be suitable analytical tools that can be used for nanostructure technologies based on nuclear tracks in thin polymer membranes. 

## Data Availability

All the data presented in this study is already available in the manuscript itself.
